# Bridging the Generational Digital Divide in the Healthcare Environment

**DOI:** 10.3390/jpm12081214

**Published:** 2022-07-26

**Authors:** Teresa Lopez de Coca, Lucrecia Moreno, Mónica Alacreu, Maria Sebastian-Morello

**Affiliations:** 1 Cátedra DeCo MICOF-CEU UCH, Universidad Cardenal Herrera-CEU, CEU Universities, 46115 Valencia, Spain; teresa.lopezperez@uchceu.es (T.L.d.C.); lmoreno@uchceu.es (L.M.); monica.alacreu@uchceu.es (M.A.); 2 Department of Pharmacy, Universidad Cardenal Herrera-CEU, CEU Universities, 46115 Valencia, Spain; 3 Embedded Systems and Artificial Intelligence Group, Universidad Cardenal Herrera-CEU, CEU Universities, 46115 Valencia, Spain

**Keywords:** digital divide, ePatient, communication technologies, telemedicine, elderly, healthcare

## Abstract

Increasing technological advances have generated a digital dependency in the population, resulting in a group of digitally excluded vulnerable people that lack basic digital skills. The aim of this study was to assess the digital divide in patients in relation to the healthcare environment. We explored the extent and effects of the digital health divide by undertaking a systematic review of the academic literature and comparing our findings with the results of a cross-sectional in-person survey answered by 881 people at four community pharmacies. In terms of the sociodemographic profile of the patients, we collected data regarding their gender, age, education level, and location (periphery or urban). The parameters evaluated were use of the internet to search for health information, use of telemedicine, use of different medical/healthcare applications, understanding explanations given by physicians regarding health, and asking pharmacists for help about newly prescribed treatments. Moreover, 168 pharmacists answered an online survey about how often they helped patients to make health center appointments or to download their COVID-19 vaccination certificate. Gender did not influence these results, but age, education level, and population location did. Those with the lowest levels of education required more help to request a health center appointment. People with high education levels and those living in an urban environment more often searched the internet for information about treatments that were new to them. Finally, people living in periphery areas received more help from their pharmacists, 60% of which said they had helped patients to download their COVID-19 vaccination certificate, with 24% of them saying they helped patients with this on a daily basis.

## 1. Introduction

In recent years, increasing technological changes have generated a digital dependency within the population. Information and communication technologies (ICTs) allow citizens to access information, work, and feel part of a social structure. The problem is that part of the population does not have the ability or knowhow to use ICTs. The term ‘digital divide’ describes having internet access problems, inadequate skills to use devices connected to the internet, or a lack of appropriate devices [1]. In addition, it may be appropriate to consider the terms ‘generation divide’ and ‘geographic divide’ as being part of the digital divide denomination. People very often search for health information on the internet. E-health resources designed for consumers, from online interventions to informative websites, require the ability to read texts, use ICTs, and adequately evaluate the content facilitated by these tools to make health decisions [2].

In this context, the term ‘telemedicine’ is currently being used to refer to the integration of ICTs and health technologies to provide healthcare and promote peoples’ wellness [3]. Telemedicine activities originated in the provision of medical support followed up with telephone consultations [4]. A wide variety of digital health technologies are now available to perform health interventions, including digital device applications (apps), SMS texts, emails, websites, chatbots, voice-calls, and videocalls, etc. Indeed, the combination of these resources, along with active user interaction, can help patients to lead healthier lives [5]. Thus, telemedicine can bring huge health benefits to people living in periphery communities, allowing them to approach healthcare services more easily. These underserved areas often lack local health centers, and additionally, this population may not have the means to travel long distances to receive the care that they need.

However, the digital divide between periphery and urban residents also extends to health technologies [6]. On the one hand, according to the Spanish National Institute of Statistics [7], the evolution of user internet access among people aged 16–74 years has increased from 78.7% in 2015 to 93.2% in 2020 with no gender differences (95.7% men and 96% women). However, this figure decreased to 31.8% of people aged over 75 years (37.5% male compared to 31.6% female), thereby highlighting the generation divide (INE). On the other hand, COVID-19 social distancing policies increased the use of virtual models of care meaning that now, more than ever, basic digital skills are essential, especially for people living in periphery areas. Therefore, additional research is still needed to assess the digital divide among patients in relation to their healthcare environments. To date, very little research has evaluated instruments used to measure digital literacy in elderly adults or its relationship to gender, education levels, or residential area as we have done in this current work.

## 2. Materials and Methods

### 2.1. Systematic Review

**Figure 1:**
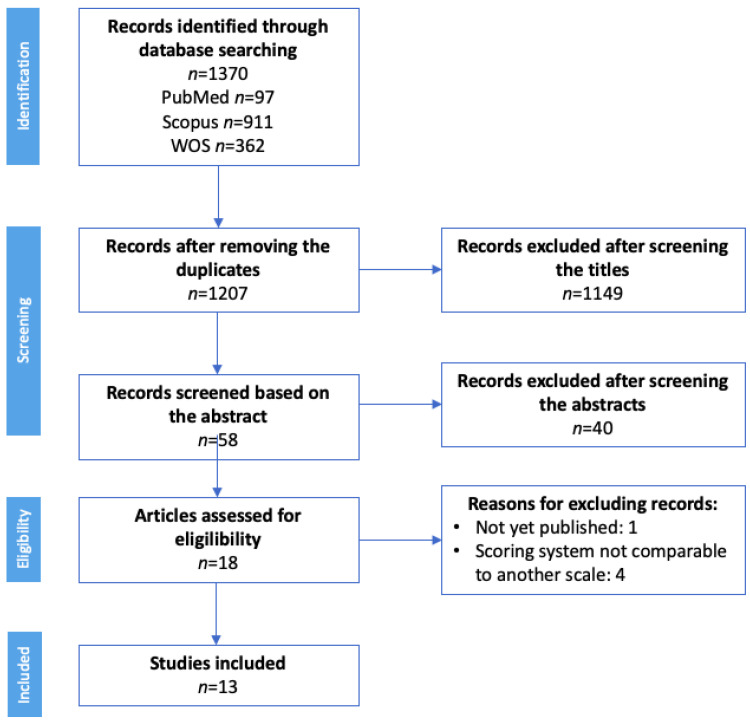
PRISMA flow diagram to describe the selection of previous studies for inclusion in this review. Abbreviations: WOS = Web of Science.

To contextualize this study, we first conducted a systematic review. The databases selected for this purpose were PubMed, Scopus, and Web of Science using the following keywords: “digital divide health” OR “digital health literacy” OR “e-patients”, using the filters shown in Table 1. This review was conducted between December 2021 and January 2022 and the search and review processes are presented in Figure 1.

### 2.2. Participant Recruitment and Data Collection

We conducted a cross-sectional study over 3 months from January to March 2022. Participants were recruited through four community pharmacies in the Valencian region (Spain), two periphery and two urbans.

All the community pharmacists involved in this work were trained by one of the authors of this current work. While patients were waiting for their medications to be dispensed, the pharmacist interviewed them, face-to-face, using an anonymized survey. To reduce bias, the survey was administered by pharmacists in practice different to the habitual ones.

The survey comprised nine questions (Annexed I, Supplementary Materials) with possible yes or no answers related to the use of health technology and consisted of the following sections: patient age and sex, education level, internet use, how they made appointments at their health center, difficulties in making medical appointments, and understanding the explanation of new treatments by physicians or pharmacists.

In addition, we created anonymous surveys for the community pharmacists in order to determine the level of assistance they had provided to help their patients download their COVID-19 certificates or make healthcare center appointments. The interview was conducted through an online questionnaire using Microsoft Forms and was distributed through pharmaceutical social networks. All the questionnaire responses were anonymized.

### 2.3. Statistical Treatment

#### 2.3.1. Calculation of the Sample Size

To estimate the average age and prevalence of the habitual use of the internet to search information among the general population, at least 683 people were needed to calculate the sample size with a confidence of 95% and an accuracy of 6%, assuming, as a reference, that 80% of the population use the internet.

#### 2.3.2. Data Protection

Information processing guarantees both the protection of the data and their security. These data were treated confidentially and lawfully and were used for the purpose for which the respondent had been informed. Thus, this work complied with the European General Data Protection Regulation (RGPD) and Organic Law 3/2018 on the Protection of Personal Data and the Guarantee of Digital Rights. The study complied with the basic principles of the Declaration of Helsinki: respect for the individual (Article 8) and recognition of their right to self-determination and their right to make informed decisions (informed consent, contained in Articles 20, 21, and 22), including participation in research, both at its beginning and throughout the work.

#### 2.3.3. Statistical Inferences

We organized the information provided by the survey participants into a Microsoft Excel spreadsheet. The statistical processing was carried out with R advanced statistical software. After configuration and outlying data of the database, we made statistical inferences to estimate the population percentage that usually uses the internet to search for information. We also sought to estimate the percentage of the population with difficulty requesting an appointment with their health center without help, and estimate the percentage who had difficulty understanding the explanation offered when they were prescribed a new treatment (95% confidence intervals).

We also searched for associations between the answers to the survey questions and the variables that defined the patient profiles; that is, their gender, age, education level, and the population location (chi-squared tests and Student *t*-tests for independent samples). A multivariate logistic regression model was then used to estimate the probability that individual participants would have difficulty in understanding new treatments according to the participant profile variables.

### 2.4. Ethical Approval

Participation in this work was anonymous. This study was reviewed and approved by the Institutional Review Board (IRB) at the CEU Cardenal Herrera University (CEEI21/260, approval date: 24 January 2022).

## 3. Results

### 3.1. Digital Divide Systematic Review

A systematic review was conducted to identify and select the most relevant articles for each keyword. The PRISMA flow diagram in shown in Figure 1 summarizes our search results and the selection process applied to all the studies we included.

A total of 1370 records was initially identified, 97 in PubMed, 911 in Scopus, and 362 in the Web of Science; 1207 records were selected after removing the duplicates. Any articles not related to the digital divide or digital health were eliminated during the title-based screening. Of the remaining 58 records, only those that included information about the digital divide, digital health, or the use of telemedicine were selected. Thus, 18 articles were finally assessed for eligibility. Four of these manuscripts were excluded because the scoring system they had employed was not comparable to other scales and another article was excluded because it had not yet been published (Figure 1).

In this systematic literature review, we found 3 groups of studies that had evaluated the following parameters (Table 2) [8,9,10,11,12,13,14,15,16,17,18,19,20]: use of the internet to search for health information, use of telemedicine, and use of different medical apps.

Table 2 provides a general summary and detailed characteristics of the studies included in this work, all of which were published between 2011 and 2020. Most studies had been conducted in the USA (6/13), while others had been conducted in Asia (4/13) or Europe (3/13). Moreover, differences between the populations included in the studies were found: eight of them had examined a population aged over 50 years, while the remaining 5 had used data without applying age constraints.

There were also disparities in the data obtained depending on the category analyzed; the results for searching for health information ranged from 16–96% in citizens aged over 50 years compared to 26.64% in studies that had covered adult population. Meanwhile, in the studies evaluating the use of health apps, 36.05–64.09% of people aged over 50 years had done so. In turn, in studies covering the younger population (>18 years), the results ranged from 0.24–70.20%. Similarly, studies that had evaluated the use of telemedicine in populations with a similar age range to this current study also showed comparable levels of divergence in their results (7.2–43.9%).

Given these variations, as well as the lack of studies carried out in Spain, we surveyed community pharmacy users to evaluate these 3 concepts in relation to patient age and education level in a similar population.

### 3.2. Cross-Sectional Study Survey Results

Our survey was answered by 881 participants and the patient profiles were studied by assessing four characteristics: gender, age, education level, and population location (periphery or urban). The results obtained are presented in Table 3 as the distribution of the responses for these 4 variables according to their individual profile. The average age of the participants was 57.1 ± 18.8 years, 62.3% of them were female and almost half of them had had a university education (47.7%).

**Figure 2:**
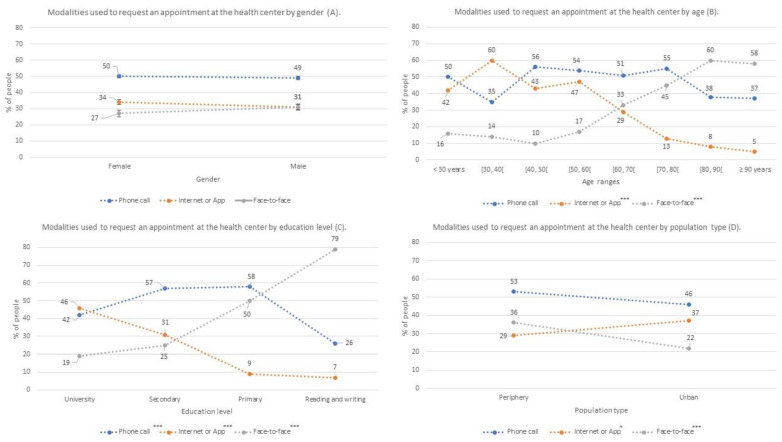
Percentages of participants who used each of the means to request an appointment at the health center (face-to-face, telephone or internet); Modalities used to request an appointment al the health center by gender (**A**); by age (**B**), by educational level (**C**) and by population type (**D**) *: *p*-value < 0.05; ***: *p*-value < 0.001.

**Figure 3:**
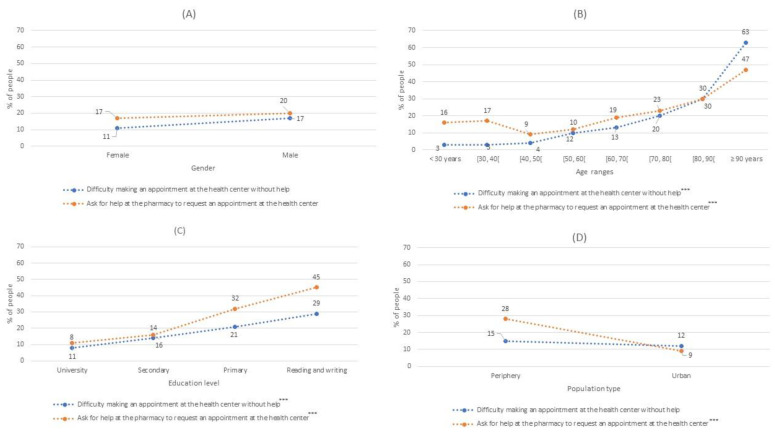
The percentages of people who had difficulty making an appointment at their health center without help or who received help at the pharmacy to make an appointment at their health center Modalities used to request an appointment al the health center by gender (**A**); by age (**B**), by educational level (**C**) and by population type (**D**). ***: *p*-value < 0.001.

**Figure 4:**
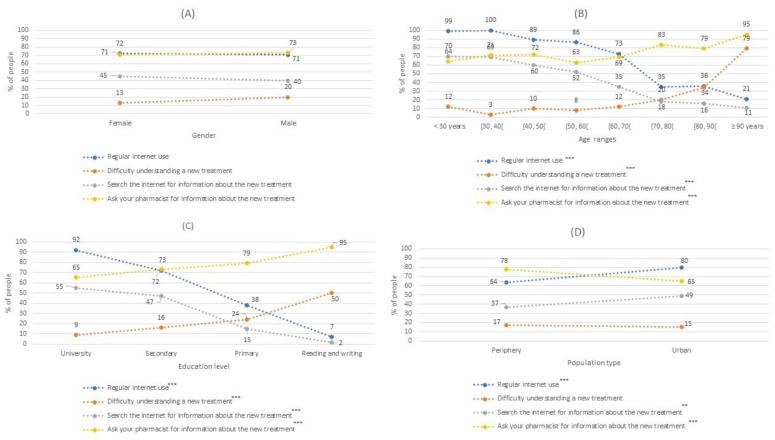
The percentage of people who habitually use the internet to search for health information, had difficulty understanding new treatments prescribed by their doctor, sought information about new treatments prescribed by their doctor, or asked their pharmacist for information about the new treatment Modalities used to request an appointment al the health center by gender (**A**); by age (**B**), by educational level (**C**) and by population type (**D**). **: *p*-value < 0.01; ***: *p*-value < 0.001.

Figure 2, Figure 3 and Figure 4 were made from Table 3. In each figure, the percentage scale is the same, to improve the comparison. In addition, asterisks have been indicated in the legends if the information represented is statistically significant, as in Table 3. It should be noted that the dashed lines of these figures represent the percentage trend between the different categories of sex, age ranges, level of education, and type of population.

The first three survey questions referred to how people requested an appointment at the health center and showed that gender does not influence these results. However, the data were influenced by age, education level, and population location. As shown in Figure 2, the method most frequently used to request an appointment (49.4% of the interviewees) was by telephone, although we did not find any significant differences for this method in terms of the patient characteristics considered. However, there were significant differences in the population regarding their use of healthcare apps in terms of age, education level, and population location. Both younger populations (Figure 2B) and those with higher education levels (Figure 2C) more often used this method to request an appointment at their health center. In contrast, elderly citizens and those with a low level of education usually went in person to their health center to request a medical appointment.

Our results indicated that between 11% and 16% of people had difficulty in requesting a health center appointment without help. Furthermore, in Question 5, 18.1% of the participants acknowledged that they had been helped by pharmacy staff to make an appointment at their health center (Figure 3). Moreover, this aid was gender-independent; likewise, the average age of those who receiving help was significantly higher than in participants who had not received this type of help (Figure 3B).

Again, participants with the two lowest levels of education required more help to request a health center appointment. It is also worth noting that significantly more people who lived in a periphery population had been helped by a pharmacist compared to urban citizens (Figure 3D). The people who had difficulty making an appointment without help at the health center were aged a mean 70.6 ± 16.4 years, had a lower level of education, and tended to live in a periphery (14.5%) rather than an urban (12%) population (Figure 3C).

Question 6 showed that 71.7% of the participants used the internet, with some 69% and 75% thought to regularly use the internet to search for information. This habit was not associated with gender but was related to age. People who often used the internet were significatively younger than those who did not use it. Likewise, this habit was significantly more frequent in people who had had a secondary or university education as well as those among an urban population (Figure 4).

As shown in Figure 4, the last three questions of the survey referred to patients’ understanding of their physician’s instructions, with 15.3% saying they did not usually understand the explanations given to them regarding newly prescribed treatments. This difficulty was significantly higher in men than in women (19.6% vs 13.3%) and the average age of those with this problem was significantly higher than those with no difficulty understanding new treatments (67.1 ± 20.4 vs 55.3 ± 17.9). Moreover, this difficulty increased in patients with lower education levels, reaching 50% among participants who were only able to read and write. In this case, the population location (periphery or urban) was not associated with this problem.

In turn, 43% of the participants said they searched for health information on the internet. This result was independent of gender, but the mean age of this group was significantly lower than those who did not search for health information on the internet (48.4 ± 17.0 vs 63.7 ± 17.3). More participants with a high school or university education, or who lived in an urban population, searched for information about new treatments prescribed to them by their physician.

On the other hand, 71.6% of the participants had asked their pharmacist for help regarding new treatments and this request for help was gender-independent. However, the average age of participants that requested help about their new treatment was significantly higher than that of citizens who did not make the same request (58.6 ± 18.9 vs 53.3 ± 18.0). People who asked for the most help had the lowest education levels (95.2%) and significantly more lived in the periphery population (78%) compared to the urban population (65.3%), as shown in Figure 4D.

After analyzing the results of the survey, we more thoroughly studied the information provided in Question 7 regarding difficulty in understanding new treatments prescribed by doctors, as well as the repercussions that this problem may have on correct adherence to medications. Thus, as shown in Table 4, we adjusted a multivariate logistic regression model to estimate the probability of difficulty in understanding new treatments based on the significant patient profile variables presented in Table 3 (gender, age, and education level). The gender variable was used as a female reference category while university education was used as the education level variable reference category. The regression model was found to have no interaction effects.

**Figure 5:**
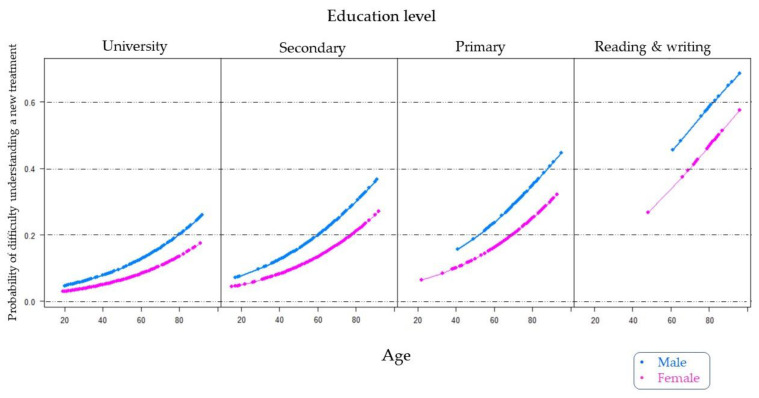
Estimation of the probability of difficulty in understanding a new treatment prescribed by the doctor according to the age of the patients, distinguishing them by their education level and gender.

Based on the logistic regression model shown in Table 4, we graphically represented a specific estimate of the probability of difficulty understanding a new treatment prescribed by a physician, separated by the education level and gender factors (Figure 5). As shown, the point estimate of the probability of male gender was higher than that for female gender, regardless of the education level. The population with the lowest level of education was much less likely to understand a new treatment than the rest of the education levels, with probabilities exceeding 0.6 in men aged over 80 years. For example, we estimated that the probability of men aged around 80 years with a university education having difficulty understanding a new treatment was close to 0.20. This same probability was found in men with a secondary education aged less than 60 years and in men with a primary education level aged approximately 50 years.

### 3.3. Pharmacist Surveys

**Figure 6:**
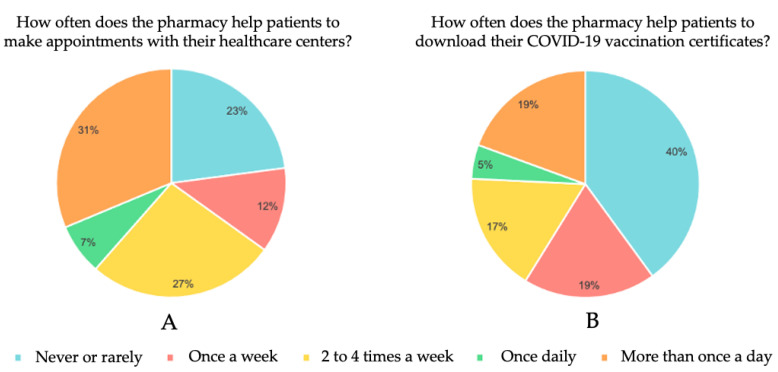
Pharmacist online survey results. How often does the pharmacy help patients to make an appointments with their healthcare centers (**A**) and to download their COVID-19 vaccination certificates (**B**).

A total of 168 pharmacists answered the online survey. Information was collected on the frequency with which they provided help to patients to make appointments with their health centers or to download their COVID-19 vaccination certificate. The results obtained are shown in Figure 6.

We observed that, because of the patients’ lack of technological knowhow, 77% of the pharmacists had helped patients at least once a week to request a healthcare center appointment; of these, 38% said they did so daily (Figure 6A). On the other hand, 60% said they helped their patients to download their COVID-19 vaccination certificates, 24% doing this for patients on a daily basis (Figure 6B).

## 4. Discussion

This is one of the first studies conducted in a Spanish population regarding the use of the internet in relation to the health environment. We compared the way patients make appointments at their health centers, whether they receive help to do so, internet use in the search for health information, understanding new prescriptions from their physician, and solicitation for help by pharmacists while considering the variables of age, gender, education level, and population location. We reviewed 13 studies and compared their results with the data we obtained by directly surveying 881 patients in person as well as through an online survey that included 168 pharmacists.

Coinciding with the results obtained in a study conducted in Switzerland [19], most patients in our work requested healthcare center appointments by telephone. No significant differences were found between the variables evaluated in relation to phone calls made to request an appointment at health centers. However, in the Swiss study, more older people requested appointments at their health center by telephone, whereas in our sample, older people tended to prefer making appointments in person. Nonetheless, our population used telephone calls to request appointments at health centers up to 5 times more often than the participants in a study conducted in Bangladesh [20]. This may be because Bangladesh is a developing country with a lower per capita GDP than Switzerland or Spain and it is more difficult to invest enough money in telecommunications resources in these kinds of emerging economies [21].

When we evaluated the use of health apps, we obtained similar use results to those from the Singapore project, with significantly lower use rates than European countries [13,14], South Korea [18], or the United States [16,17]. However, Switzerland [19] does not use these apps (0.24%). On the one hand, no significant differences were found in terms of gender when evaluating the use of healthcare apps, which agreed with studies conducted in Europe [13,14] and the United States [16,17]. Finally, the study conducted in Singapore showed that men used health apps more often than women [15].

On the other hand, participant age was inversely proportional to the use of health apps, whereby the older the interviewees, the less they used this resource [14,16,19]. In addition, our results in relation to education levels also agreed with other studies with the situation changing at older ages. People who had completed their university degrees longer ago would have completed courses that did not provide adequate ICT skills, thereby leading to a significant increase in the demand for in-person care resources in their old age [8,14,15,18,19,22].

In this work, patients were asked if they were able to make an appointment at their health center without help or if they were usually helped by their community pharmacy to do so; we consider this factor to be especially important in terms of preventive health interventions. Indeed, the number of patients who needed help to request a medical appointment (13.3%) was related to the figure for the help provided by pharmacists to make an appointment at a health center (18.1%). These results were lower than those obtained in parallel with the pharmacists’ survey responses, in which 77% of the pharmacists interviewed said that they helped patients at least once a week to make an appointment at their health center, with 38% saying that they did so daily.

Of note, the average age of patients who needed assistance to request medical appointments was significantly higher than those who did not. The opposite was observed for education levels, with patients with lower levels of education requiring more help to make an appointment with their physician. According to the INE, in 2020, 93.2% of the population aged between 16 and 74 years accessed the internet, while only 31.8% of people aged over 75 years used the internet, without gender differences in either case [7]. In our survey, we estimated that between 69% and 75% of people use the internet regularly, with this percentage decreasing at older ages or with lower education levels. Moreover, according to pharmacists, our patient cohort appeared to use the internet less than the average population in Spain [7].

The last part of our survey collected data about whether patients searched for information related to new treatments they had been prescribed. Namely, if they had understood their physician’s explanation, had consulted a pharmacist, or had searched for the new medication on the internet. Most of the interviewed population (84.3%) said that they had understood the explanation of the treatments given to them by their physician, although this trend changed starting from 70 years, with the role of pharmacists thereafter becoming more relevant in improving patient comprehension.

This change might be because of a poorer general understanding of the physician explanations from this age, with some studies attributing it to the embarrassment that patients may feel related to not understanding the doctor when at older ages [23]. This segment of the population has a higher incidence of chronic disease, polypharmacy, and tends to have more difficulty using the internet and looking for health information [22,24]. In this current work, younger participants preferred to search the internet for information, while older patients more often consulted their physician or pharmacist. Less than half the citizens interviewed reported using the internet to search for health information and their mean age was significantly lower (48.4 ± 17 years) than those who did not use it (63.7 ± 17.3 years). Indeed, 26.64% of our population used this resource, representing almost twice as many as the European average [13], and almost 3 times as much as the South Korean population at 16% [11].

However, 67.3–74.79% of the Spanish population used the internet to search for information about new medications, fewer than in the North American population [9,10,12]. Of note, all these studies investigated populations aged over 50 years, except the European one, which considered a population with a similar age range to ours. Importantly, use of the internet to search for information about new treatments was proportional to the level of patient education and was inversely proportional to age, as also confirmed by studies conducted by other researchers [8,13,25].

In addition, there was a gender division regarding internet use to search for health information at older ages. Some researchers described that, among older adults, males more often used the internet to search for medical information, but this was because they were more likely to use the internet in general [15]. There is currently no gender divide regarding internet use at younger ages in Spain [7] and so it has been suggested that when the population gets older, internet use will increase and will be prevalent even among the future elderly population [22,26], with gender and generational divides perhaps disappearing.

The use of the internet for healthcare purposes is considered an important solution to adequately meet the complex care needs of people with several illnesses [27]. However, it is important to highlight the existence of ‘fake news’ which also affects the health area. These news feeds often contain inaccurate information and can promote distrust of healthcare interventions among the population.

Bridging the gap is necessary to ensuring that digital health tools are used correctly and competently in practice. Identification of patients without devices or internet is the first step. Digital health tools will only be effective once a common knowledge base exists, so, building an accessible, easily navigable solution and educating users is necessary.

Moreover, it will be important also to reinforce and improve patient–physician relationships in the future. Professionals should try to use accessible language, clear up any doubts their patients may have, and provide them with reliable sources of health information they can search for on the internet [22].

The results of the last part of our survey showed that eHealth literacy levels must still be increased, especially among the elderly population, in order to avoid mismanagement of health information and direct it more towards reliable sources. Therefore, more studies will be required to understand differences in the populations under study. In addition, as patients get older, their medication needs tend to increase, as shown in Figure 5. Finally, part of the information collected in the surveys cannot be compared with other results because, in many cases, previous work did not examine these questions. The sample we studied was also limited to a single geographic region with specific health management characteristics. Lastly, we collected extensive information from patients and limited data from pharmacists but did not consult physicians. Therefore, future projects should study all these healthcare system components.

## 5. Conclusions

The technological progress observed in recent years has increased the digital divide according to age, especially in patients aged over 70 years. Older patients are more reticent in the use of new technologies such as health apps and because of the difficulties new technologies cause them. They instead prefer to request appointments in person or through their pharmacists. The digital divide in terms of gender continues to be widespread among the older population; they experience greater difficulty in making appointments at their health center or understanding new treatments, while this gender divide is considerably reduced in younger generations. Finally, because of the limited time patients spend in medical consultations, those with lower levels of digital literacy usually need help to understand new treatments prescribed to them. Among others, pharmacists are offering this help.

## Figures and Tables

**Table 1:** Parameters of the systematic academic literature review.

Database	Filters
PubMed	Last 5 years Humans Type of document: case report, classical article, clinical study, or randomized controlled trial
Scopus	Last 5 years English or Spanish Area of research: medicine, health professions, pharmacology, toxicology and pharmaceutics, or multidisciplinary
Web of Science	2016–2022 English or Spanish Humans Type of document: article, clinical trial, case report, non-review articles

The filters we applied to the search strategy used for different databases.

**Table 2:** Evaluation of the use of the internet to search for health information, use of medical apps, and telemedicine. All the data from these studies were collected through questionnaires.

Article	Evaluation	Data Collection Period	Individuals (*n*)	Male	Female	Pathology	Age	Results	Comments
Yoon H, 2020, [8] USA	Searching for health information on the internet	2011–2016	107,500	40.10%	59.90%	Any patient regardless of their condition	>60	60.2% in 2011 vs. 67.3% in 2016	35.9% of the questionnaire totals were from patients aged >75 years.
Price-Haywood EG, 2017, [9] USA	Searching for health information on the internet	2015–2016	137 used an app vs. 110 non app users	30% used an app vs. 42% non app users *	70% used an app vs. 58% non app users	Hypertension and/or diabetes	>50	Internet search: 96% app users vs. 56% non app users	78.14% of the population interviewed *
Choi EY, 2020, [10] USA	Searching for health information on the internet	2016	5914	40.43% *	59.57% *	Any patient regardless of their condition	>50	74.79%	The difference in gender was not statistically significant but did show the generational divide
Park S, 2020, [11] South Korea	Searching for health information on the internet	2017	1919	68.37% *	31.63% *	Diabetics	>65	16% *	17.4% of the respondents only used the internet to send or receive text messages
Vollbrecht H, 2020, [12] USA	Searching for health information on the internet	2020	178	47%	53%	Any patient regardless of their condition	Median 55 years old	67%	84% of interviewees used the internet
Alvarez-Galvez J, 2020, [13] 28 European countries	Searching for health information on the internet	2014	26,566 (1000 from Spain)	65.40%	77%	Any patient regardless of their condition	>18	26.64%	7.56% visited official health websites
	Use of health apps							25.77% (9.69% male and 16.08% female) *	
Lämsä E, 2017, [14] Finland	Use of health apps	2015	1288	25% *	75% *	Any patient regardless of their condition	18–93	62.10%	60–70% aged 18–74 years; 38.3% aged >75 years.
Ang S, 2020, [15] Singapore	Use of health apps	2016–2017	3966	48.34%.	51.66%	Any patient regardless of their condition	>60	36.05% (no significant differences between the genders)	8.18% had problems using the app studied
Walker DM, 2019, [16] USA	Use of health apps	2017–2018	848	39%	61%	Any patient regardless of their condition	>18	70.20%	This article showed how older patients needed more tutorials to use health apps
Hung LY, 2020, [17] USA	Use of health apps	2018	50,904,732	45.06% *	54.94% *	Any patient regardless of their condition	>65	43.88% (44.47% male and 43.40% female) *	Scheduled medical appointments via the internet
Lee M, 2020, [18] South Korea	Use of health apps	2018	323	38.08% *	61.92% *	Any patient regardless of their condition	>50	64.09% * (38.2% male and 61.8% female)	12.1% aged >70 years and 87.9% aged <70 years
Mettler AC., 2021, [19] Switzerland	Use of health apps	2018	417	44.60%	55.40%	Any patient regardless of their condition	29–49	0.24% *	84.06% minor health issues, 15.93% serious health issues, 72.7% phone calls, 26.8% internet resource, 0.5% phone app *
	Use of telemedicine							43.9% (53.5% male and 46.5% female)	
Ahmed T, 2019, [20] Bangladesh	Use of telemedicine	2013–2014	854	28.10% *	71.90% *	Any patient regardless of their condition	25–54	7.20%	64.7% minor health issues and 35.3% with serious health issues

* These data were converted to percentages.

**Table 3:** Survey responses based on the participants’ gender, age, education level, and population location.

SURVEY QUESTIONS	TOTAL *n* = 881 (%; CI(95%))	Association with Gender			Association with Age		Association with Level of Education					Association with Population Type		
		GENDER		*p*-Value	AGE	*p*-Value	LEVEL OF EDUCATION				*p*-Value	POPULATION TYPE		*p*-Value
		Female *n* = 549 (62.3%)	Male *n* = 332 (37.7%)		$\bar{x}\pm s$ 57.1 ± 18.8		Read & write *n* = 42 (4.8%)	Primary *n* = 144 (16.3%)	Secondary *n* = 319 (36.2%)	University *n* = 376 (47.7%)		Periphery *n* = 440 (50.0%)	Urban *n* = 441 (50.0%)	
1. Do you request an appointment at the health center in person? No Yes	628 (71.3; [68.2, 74.2]) 253 (28.7; [25.8, 31.8])	400 (72.9) 149 (27.1)	228 (68.7) 104 (31.3)	0.192 ^a^	53.1 ± 17.8 67.1 ± 17.5	<0.001 ^b^ ***	9 (21.4) 33 (78.6)	72 (50.0) 72 (50.0)	241 (75.5) 78 (24.5)	306 (81.4) 70 (18.6)	<0.001 ^a^ ***	283 (64.3) 157 (35.7)	345 (78.2) 96 (21.8)	<0.001 ^a^ ***
2. Do you request an appointment at the health center by phone? No Yes	446 (50.6; [47.3, 53.9]) 435 (49.4; [46.1, 52.7])	275 (50.1) 274 (49.9)	171 (51.5) 161 (48.5)	0.728 ^a^	57.4 ± 19.6 56.9 ± 17.9	0.703 ^b^	31 (73.8) 11 (26.2)	60 (41.7) 84 (58.3)	136 (42.6) 183 (57.4)	219 (58.2) 157 (41.8)	<0.001 ^a^ ***	208 (47.3) 232 (52.7)	238 (54.0) 203 (46.0)	0.051 ^a^
3. Do you request an appointment at the health center online or through the app? No Yes	592 (67.2; [64.0, 70.2]) 289 (32.8; [29.8, 36.0])	362 (65.9) 187 (34.1)	230 (69.3) 102 (30.7)	0.366 ^a^	61.0 ± 18.8 49.1 ± 16.1	<0.001 ^b^ ***	39 (92.9) 3 (7.1)	131 (91.0) 13 (9.0)	219 (68.7) 100 (31.3)	203 (54.0) 173 (46.0)	<0.001 ^a^ ***	314 (71.4) 126 (28.6)	278 (63.0) 163 (37.0)	0.010 ^a^ *
4. Are you able to make an appointment without help at the health center? No Yes	117 (13.3 [11.2, 15.7]) 764 (86.7; [84.3, 88.8])	62 (11.3) 487 (88.7)	55 (16.6) 277 (83.4)	0.031 ^a^ *	70.6 ± 16.4 55.1 ± 18.3	<0.001 ^b^ ***	12 (28.6) 30 (71.4)	30 (20.8) 114 (79.2)	45 (14.1) 274 (85.9)	30 (8.0) 346 (92.0)	<0.001 ^a^ ***	64 (14.5) 376 (85.5)	53 (12.0) 388 (88.0)	0.277 ^a^
5. To make an appointment, were you helped by the pharmacy? No Yes	722 (82.0; [79.3, 84.4]) 159 (18.1; [15.6, 20.7])	455 (82.9) 94 (17.1)	267 (80.4) 65 (19.6)	0.367 ^a^	55.8 ± 18.3 63. ± 19.8	<0.001 ^b^ ***	23 (54.8) 19 (45.2)	98 (68.1) 46 (31.9)	268 (84.0) 51 (16.0)	333 (88.6) 43 (11.4)	<0.001 ^a^ ***	319 (72.5) 121 (27.5)	403 (91.4) 38 (8.6)	<0.001 ^a^ ***
6. Do you use the internet? No Yes	249 (28.3; [25.4, 31.3]) 632 (71.7; [68.7, 74.6])	154 (28.1) 395 (71.9)	95 (28.6) 237 (71.4)	0.877 ^a^	72.3 ± 12.5 51.1 ± 17.4	<0.001 ^b^ ***	39 (92.9) 3 (7.1)	89 (61.8) 55 (38.2)	89 (27.9) 230 (72.1)	32 (8.5) 344 (91.5)	<**0.001** ^a^ ***	159 (36.1) 281 (63.9)	90 (20.4) 351 (79.6)	<0.001 ^a^ ***
7. When a new treatment is prescribed, do you understand your physician’s explanation? No Yes	138 (15.7; [13.4, 18.2]) 743 (84.3; [81.8, 86.6])	73 (13.3) 476 (86.7)	65 (19.6) 267 (80.4)	0.017 ^a^ *	67.1 ± 20.4 55.3 ± 17.9	<0.001 ^b^ ***	21 (50.0) 21 (50.0)	34 (23.6) 110 (76.4)	50 (15.7) 269 (84.3)	33 (8.8) 343 (91.2)	<0.001 ^a^ ***	73 (16.6) 367 (83.4)	65 (14.7) 376 (85.3)	0.460 ^a^
8. When a new treatment is prescribed, do you search on internet for information about it? No Yes	502 (57.0; [53.7, 60.2]) 379 (43.0; [39.8, 46.3])	303 (55.2) 246 (44.8)	199 (59.9) 133 (40.1)	0.182 ^a^	63.7 ± 17.3 48.4 ± 17.0	<0.001 ^b^ ***	41 (97.6) 1 (2.4)	122 (84.7) 22 (15.3)	169 (53.0) 150 (47.0)	170 (45.2) 206 (54.8)	<0.001 ^a^ ***	276 (62.7) 164 (37.3)	226 (51.2) 215 (48.8)	0.001 ^a^ **
9. When a new treatment is prescribed, do you ask your pharmacist for information about it? No Yes	250 (28.4; [25.5, 31.4]) 631 (71.6; [68.6, 74.5])	161 (29.3) 388 (70.7)	89 (26.8) 243 (73.2)	0.441 ^a^	53.3 ± 18.0 58.6 ± 18.9	<0.001 ^b^ ***	2 (4.8) 40 (95.2)	30 (20.8) 114 (79.2)	86 (27.0) 233 (73.0)	132 (35.1) 244 (64.9)	<0.001 ^a^ ***	97 (22.0) 343 (78.0)	153 (34.7) 288 (65.3)	<0.001 ^a^ ***

^a^: *p*-value of the Chi-square test; ^b^: *p*-value of Test T for independent samples; Significant *p*-values are indicated in bold. *: *p*-value < 0.05; **: *p*-value < 0.01; ***: *p*-value < 0.001. IC (95%): Confidence Interval at 95%.

**Table 4:** Logistical regression model for difficulty in understanding a newly prescribed treatment adjusted for age, gender, and education level.

Variable	β_i_	*SD*	Wald	d.f.	*p*-Value	Exp(β_i_)	95% CI	
							UL	LL
Intercept	−4.033	−0.422	−9.55	1	<0.001 ***	0.018	0.007	0.039
Age	0.027	0.006	4.260	1	<0.001 ***	1.028	1.015	1.041
Gender (male)	0.475	0.197	2.407	1	0.016 *	1.608	1.091	2.367
Education level (secondary)	0.530	0.245	2.167	1	0.030 *	1.700	1.056	2.765
Education level (primary)	0.754	0.286	2.640	1	0.008 **	2.126	1.214	3.730
Education level (reading and writing)	1.718	0.389	4.419	1	<0.001 ***	5.576	2.606	12.034

β_i_: model coefficients; *SD*: standard deviation of the coefficients; d.f.: degrees of freedom; Exp(βi): odds ratio; UL: upper limit of the 95% confidence interval for the expected odds ratio; LL: lower limit of the 95% confidence interval for the expected odds ratio; *: *p*-value < 0.05; **: *p*-value < 0.01; ***: *p*-value < 0.001.

## Supplementary Materials

The following supporting information can be downloaded at: https://www.mdpi.com/article/10.3390/jpm12081214/s1, Survey questions.
