# Supplementary material for: Bridging the Generational Digital Divide in the Healthcare Environment
Source: J Pers Med. 2022 Jul 26;12(8):1214. doi: 10.3390/jpm12081214 (PMC9394326; doi:10.3390/jpm12081214)
Supplement: Supplementary file 1 [file jpm-12-01214-s001.zip › jpm-1808352-supplementary.pdf]

AGE: **GENDER:** **M** ☐ **F** ☐

Study level	☐ Read and write	
	☐ Primary	
	☐ Secondary	
	☐ University	
How do you request an appointment at the health center?	☐ In person	
	☐ By phone	
	☐ By internet or App	
Do you use internet?	Yes	No
Are you able to make an appointment without help at the health center?	Yes	No
To make an appointment, were you helped by the pharmacy?	Yes	No
**When a new treatment is prescribed...**		
Do you understand your physician's explanation?	Yes	No
Do you search on internet for information about it?	Yes	No
Do you ask your pharmacist for information about it?	Yes	No
